# Supplementary material for: Empirical methods for controlling false positives and estimating confidence in ChIP-Seq peaks
Source: BMC Bioinformatics. 2008 Dec 5;9:523. doi: 10.1186/1471-2105-9-523 (PMC2628906; doi:10.1186/1471-2105-9-523)
Supplement: Additional File 1 — A variety of html documents from the USeq web site detailing the available applications, their best usage, output file type descriptions, command line menus, etc. [file 1471-2105-9-523-S1.zip › USeqUserGuides/JavaDoc.zip!/JavaDoc/edu/utah/seq/data/class-use/PointData.html]

Uses of Class edu.utah.seq.data.PointData


|  |  |  |
| --- | --- | --- |
| **Overview** \| **Package** \| **Class** \| **Use** \| **Tree** \| **Deprecated** \| **Index** \| **Help** | |  |
| PREV   NEXT | **FRAMES**    **NO FRAMES**     **All Classes** |  |


---


## **Uses of Class edu.utah.seq.data.PointData**

| Packages that use PointData | |
| --- | --- |
| **edu.utah.seq.data** | Helper classes for working with binary PointData, EnrichedRegions, etc. |
| **edu.utah.seq.parsers** | Parsers for converting sequencing data into binary PointData, xxx.bar.zip files. |

Uses of PointData in edu.utah.seq.data

| Methods in edu.utah.seq.data that return PointData | |
| --- | --- |
| `static PointData` | `PointData.combinePointData(java.util.ArrayList<PointData> pdAL, boolean nullOriginalPosVal)`             Combines without summing the scores, thus duplicate positions are likely. |
| `static PointData` | `Point.extractPositionScores(Point[] p)` |
| `PointData` | `HeatMapMakerPosNeg.makeHeatMapPositionValues(SmoothingWindow[] win)`             Makes a stair step heat map from an array of windows in bar format. |
| `PointData` | `HeatMapMaker.makeHeatMapPositionValues(SmoothingWindow[] windows, boolean oneDecimal)`             Returns a PointData object with loaded position and values representing a top score heatmap/ stairstep. |
| `static PointData` | `PointData.mergePointData(java.util.ArrayList<PointData> pdAL, boolean nullOriginalPosVal)`             Merges PointData, nulls originals positions and scores, sets strand to '.' if mixed strand. |
| `PointData[]` | `PointData.split(boolean[] b)`             Splits this.PointData's positions and scores based on the boolean[]. |

| Methods in edu.utah.seq.data that return types with arguments of type PointData | |
| --- | --- |
| `java.util.HashMap<java.lang.String,java.util.ArrayList<PointData>>` | `PointData.fetchPointData(java.io.File directory)`             Loads a directory of xxx.bar PointData files into a HashMap where the key = |
| `static java.util.HashMap<java.lang.String,PointData>` | `PointData.fetchPointData(java.io.File directory, java.io.File mergeDirectory, boolean sumScores)`             Fetches PointData from a directory. |
| `static java.util.HashMap<java.lang.String,PointData>` | `PointData.fetchRandomObservations(java.util.HashMap<java.lang.String,PointData> pd, int numWanted)`             Lots of work to fetch random observation. |
| `static java.util.HashMap<java.lang.String,PointData>` | `PointData.mergePointData(java.util.HashMap<java.lang.String,PointData>[] hashes, boolean sumScores, boolean nullOriginalPosVal)`             Merges a HashMap by either summing same position scores or simply concatinating then sorting the arrays. |
| `static java.util.HashMap<java.lang.String,PointData>` | `PointData.subSamplePercent(java.util.HashMap<java.lang.String,PointData>[] pd, int numPos)`             Will draw the numPos wanted from the array of PointData sampling randomly within but equally throughout. |

| Methods in edu.utah.seq.data with parameters of type PointData | |
| --- | --- |
| `static int[]` | `PointData.findMinMaxPosition(PointData[] pd)`             Finds the min and maximum positions from all of the position arrays. |

| Method parameters in edu.utah.seq.data with type arguments of type PointData | |
| --- | --- |
| `static PointData` | `PointData.combinePointData(java.util.ArrayList<PointData> pdAL, boolean nullOriginalPosVal)`             Combines without summing the scores, thus duplicate positions are likely. |
| `static java.util.HashMap<java.lang.String,PointData>[]` | `PointData.divide(java.util.HashMap<java.lang.String,PointData> pd, int splitIn)`             Randomly divides data. |
| `static java.util.HashMap<java.lang.String,PointData>` | `PointData.fetchRandomObservations(java.util.HashMap<java.lang.String,PointData> pd, int numWanted)`             Lots of work to fetch random observation. |
| `static PointData` | `PointData.mergePointData(java.util.ArrayList<PointData> pdAL, boolean nullOriginalPosVal)`             Merges PointData, nulls originals positions and scores, sets strand to '.' if mixed strand. |
| `static java.util.HashMap<java.lang.String,PointData>[]` | `PointData.split(java.util.HashMap<java.lang.String,PointData> pd)`             Splits the data in half using random sampling. |
| `static boolean` | `PointData.subSample(java.util.HashMap<java.lang.String,PointData> pd, int numPos)`             Reduces the number of observations to the specified number by random selection. |
| `static int` | `PointData.totalObservations(java.util.HashMap<java.lang.String,PointData> pd)`             Counts the total number of observations. |
| `static boolean` | `PointData.writePointData(java.util.HashMap<java.lang.String,PointData> pd, java.io.File saveDirectory)`             Writes zip compressed 'chromosome\_strand\_.bar.zip' files into the saveDirectory. |

Uses of PointData in edu.utah.seq.parsers

| Methods in edu.utah.seq.parsers that return PointData | |
| --- | --- |
| `PointData` | `Tag2Point.loadPositionValues(int[] sortedPositions)` |

---


|  |  |  |
| --- | --- | --- |
| **Overview** \| **Package** \| **Class** \| **Use** \| **Tree** \| **Deprecated** \| **Index** \| **Help** | |  |
| PREV   NEXT | **FRAMES**    **NO FRAMES**     **All Classes** |  |


---
